# A core outcome set for studies evaluating the effectiveness of prepregnancy care for women with pregestational diabetes

**DOI:** 10.1007/s00125-017-4277-4

**Published:** 2017-04-13

**Authors:** Aoife M. Egan, Sander Galjaard, Michael J. A. Maresh, Mary R. Loeken, Angela Napoli, Eleni Anastasiou, Eoin Noctor, Harold W. de Valk, Mireille van Poppel, Marie Todd, Valerie Smith, Declan Devane, Fidelma P. Dunne

**Affiliations:** 1 Galway Diabetes Research Centre, Department of Medicine, National University of Ireland Galway, Galway, Ireland; 2 Department of Obstetrics and Gynaecology, Division of Obstetrics and Prenatal Medicine, Erasmus MC, University Medical Centre Rotterdam, ‘s-Gravendijkwal 230, 3015 CE Rotterdam, the Netherlands; 3 Department of Obstetrics, St Mary’s Hospital, Central Manchester University Hospitals NHS Foundation Trust, Manchester Academic Health Science Centre, Manchester, UK; 4 Section of Islet Cell and Regenerative Biology, Joslin Diabetes Center, Boston, MA USA; 5 Department of Medicine, Harvard Medical School, Boston, MA USA; 6 Department of Clinical and Molecular Medicine, S. Andrea University Hospital, Sapienza, University of Rome, Rome, Italy; 7 Department of Endocrinology & Diabetes Center Alexandra Hospital, Athens, Greece; 8 Department of Endocrinology, University Hospital Limerick, Limerick, Ireland; 9 Department of Internal Medicine, University Medical Centre Utrecht, Utrecht, the Netherlands; 10 Institute of Sport Science, University of Graz, Graz, Austria; 11 Department of Public and Occupational Health, Amsterdam Public Health Research Institute, VU University Medical Centre, Amsterdam, the Netherlands; 12 Department of Medicine, Mayo University Hospital, Castlebar, Ireland; 13 School of Nursing & Midwifery, Trinity College Dublin, Dublin, Ireland; 14 School of Nursing & Midwifery, National University of Ireland Galway, Galway, Ireland; 15 Health Research Board – Trials Methodology Research Network (HRB-TMRN), Galway, Ireland

**Keywords:** Clinical diabetes, Healthcare delivery, Other techniques, Pregnancy

## Abstract

**Aims/hypothesis:**

The aim of this study was to develop a core outcome set (COS) for trials and other studies evaluating the effectiveness of prepregnancy care for women with pregestational (pre-existing) diabetes mellitus.

**Methods:**

A systematic literature review was completed to identify all outcomes reported in prior studies in this area. Key stakeholders then prioritised these outcomes using a Delphi study. The list of outcomes included in the final COS were finalised at a face-to-face consensus meeting.

**Results:**

In total, 17 outcomes were selected and agreed on for inclusion in the final COS. These outcomes were grouped under three domains: measures of pregnancy preparation (*n* = 9), neonatal outcomes (*n* = 6) and maternal outcomes (*n* = 2).

**Conclusions/interpretation:**

This study identified a COS essential for studies evaluating prepregnancy care for women with pregestational diabetes. It is advocated that all trials and other non-randomised studies and audits in this area use this COS with the aim of improving transparency and the ability to compare and combine future studies with greater ease.

**Electronic supplementary material:**

The online version of this article (doi:10.1007/s00125-017-4277-4) contains peer-reviewed but unedited supplementary material, which is available to authorised users.

## Introduction

Women with pre-existing diabetes during pregnancy, also referred to as pregestational diabetes, have an increased risk of adverse pregnancy outcomes including congenital anomalies, stillbirth and perinatal mortality [1, 2]. It is well established that these risks can be reduced by attendance at prepregnancy care [3, 4]. Prepregnancy care describes the targeted support and additional care offered to women who are planning pregnancy [4]. It typically involves regular review by a multidisciplinary diabetes team in a dedicated outpatient clinic. In general, women attending prepregnancy care undergo a full medication review, assessment and treatment of diabetes complications as required and optimisation of glycaemic control. However, there is not an agreed proforma for delivery of this care and, while many groups have reported positive benefits associated with specific programmes, the outcomes reported are varied [3–5]. This inconsistency raises concern for outcome selection bias, makes meaningful comparison between studies difficult and limits the ability to combine the findings of individual studies into summary estimates [6]. One approach to overcome this lack of uniformity is to develop a core outcome set (COS) or an agreed set of outcomes. The goal is that the COS will be collected and reported in all studies that report a specific clinical condition [7]. It represents a minimum that should be collected and reported, but does not restrict researchers from adding additional outcomes at their discretion. The development of a COS across multiple disciplines is supported by the Core Outcome Measures in Effectiveness Trials (COMET) initiative, which brings together interested researchers and minimises duplication of work [7, 8]. The Core Outcome Set – STAndards for Reporting (COS-STAR) statement aims to standardise COS reporting for the benefit of all users [9]. More specifically, in the field of women’s health, the editors of over 50 journals recently endorsed the Core Outcomes in Women’s Health (CROWN) initiative [10]. Launched in 2014, this initiative has several aims including encouraging COS development and facilitating effective dissemination of manuscripts.

The aim of this study was to develop a COS for trials and other studies evaluating the effectiveness of prepregnancy care for women with pregestational diabetes mellitus.

## Methods

This study is registered in the COMET database and a detailed study protocol was published previously [6, 11]. Ethical approval for the study was obtained from the Galway University Hospital ethics committee. The COS was developed by completing a systematic literature review to identify all outcomes reported in prior studies in this area. Key stakeholders then prioritised these outcomes using a Delphi study, providing a preliminary COS. The list of outcomes included in the COS were finalised at a face-to-face consensus meeting.

### Systematic review

The protocol for the search strategy has been published previously [6]. The following databases were searched for relevant studies: MEDLINE, EMBASE, Web of Science, the Cochrane Library and the Cumulative Index to Nursing and Allied Health Literature (CINAHL). Clinicaltrials.gov was searched for relevant ongoing trials. We included prospective cohort studies, case–control studies, randomised trials and systematic reviews published in the English language that evaluated prepregnancy care for women with diabetes. Two reviewers (F. P. Dunne and A. M. Egan) independently assessed the titles and abstracts of identified studies. Full texts of studies meeting the inclusion criteria (or in the case of uncertainty regarding inclusion) were retrieved and consensus was achieved on inclusion status. The reviewers then extracted the following data from each study: study design, author details, year and journal of publication, targeted condition, intervention under investigation, each outcome specified in methods or findings, definition and method of collection used (if available) and time points or periods of outcome measurement. Following review by F. P. Dunne, A. M. Egan, D. Devane and three additional key stakeholders known as the study advisory group (SAG), outcomes were grouped under three domains: measures of pregnancy preparation, neonatal outcomes and maternal outcomes.

### Delphi study

Questionnaires were completed online using SurveyMethods software (www.surveymethods.com; accessed 21 March 2017). Participants were recruited from within the following groups: women with diabetes, midwives, obstetricians, paediatricians/neonatologists, policy makers, other service providers and researchers with an interest in diabetes in pregnancy. We sent an email inviting participation to the list managers in the following organisations: International Association of the Diabetes and Pregnancy Study Groups (IADPSG), Diabetes Ireland (DI), Irish Endocrine Society (IES), International Federation of Gynecology and Obstetrics (FIGO), European Board and College of Obstetrics and Gynaecology (EBCOG), Irish Nutrition and Dietetic Institute (INDI), Irish Institute of Obstetricians and Gynaecologists, Saolta Healthcare Group (Ireland), EASD and the Diabetic Pregnancy Study Group (DPSG) of the EASD. Snowball sampling was encouraged (i.e. participants were asked to forward the invitation to others whom they regarded as having the required expertise).

In the round 1 survey instrument, outcomes identified following the systematic review were presented to participants, grouped by domain. Related outcomes were presented alongside each other (e.g. measures of glucose control during pregnancy). Participants were asked to rate each one on a nine point Likert-type scale with higher values representing increased importance for inclusion in the COS. Participants had an opportunity to list additional outcomes for consideration in subsequent rounds of development. Study participants gave informed consent prior to the submission of any answers and the following information was also requested: name, email address, sex, stakeholder group and country of residence. The results of round 1 were summarised using descriptive statistics. All outcomes were carried forward to round 2 including additional outcomes suggested by participants in round 1. Participants who responded to round 1 were invited to participate in round 2. In round 2, they were shown their scores from round 1 and presented with the distribution of scores for each outcome per stakeholder group. Participants were invited to re-score the outcomes. All outcomes that had a median score of ≥4 for any group were carried forward to round 3. Participants who completed rounds 1 and 2 were invited to complete round 3. Each participant was presented with their round 2 scores and the distribution of scores for each outcome as per stakeholder group. Participants were asked to re-score the outcomes. Outcomes were classified as ‘consensus in’ (≥70% participants scoring as 7–9 and <15% scoring as 1–3), ‘consensus out’ (≥70% scoring as 1–3 and <15% scoring as 7–9) or ‘no consensus’ (anything else) [12].

### Consensus meeting

This final phase involved a face-to-face meeting with key stakeholders representing a range of views of service users, clinicians and researchers. The meeting was chaired by D. Devane, who did not vote at the meeting. Outcomes classified as ‘consensus in’ or ‘no consensus’ were presented to the group along with the response results from round 3 of the Delphi study. There was opportunity for open discussion and for combining or modifying individual outcomes. Participants were asked to vote on each listed outcome as ‘yes’ or ‘no’ for inclusion in the final COS. Outcomes for which ≥70% of participants voted ‘yes’ were carried forward to a further discussion and a second, final, vote. An outcome was included in the COS when ≥70% participants voted ‘yes’ in this final vote. Participants used Poll Everywhere, a downloadable application, to place their vote anonymously (www.polleverywhere.com; accessed 21 March 2017).

## Results

### Systematic review

**Fig. 1:**
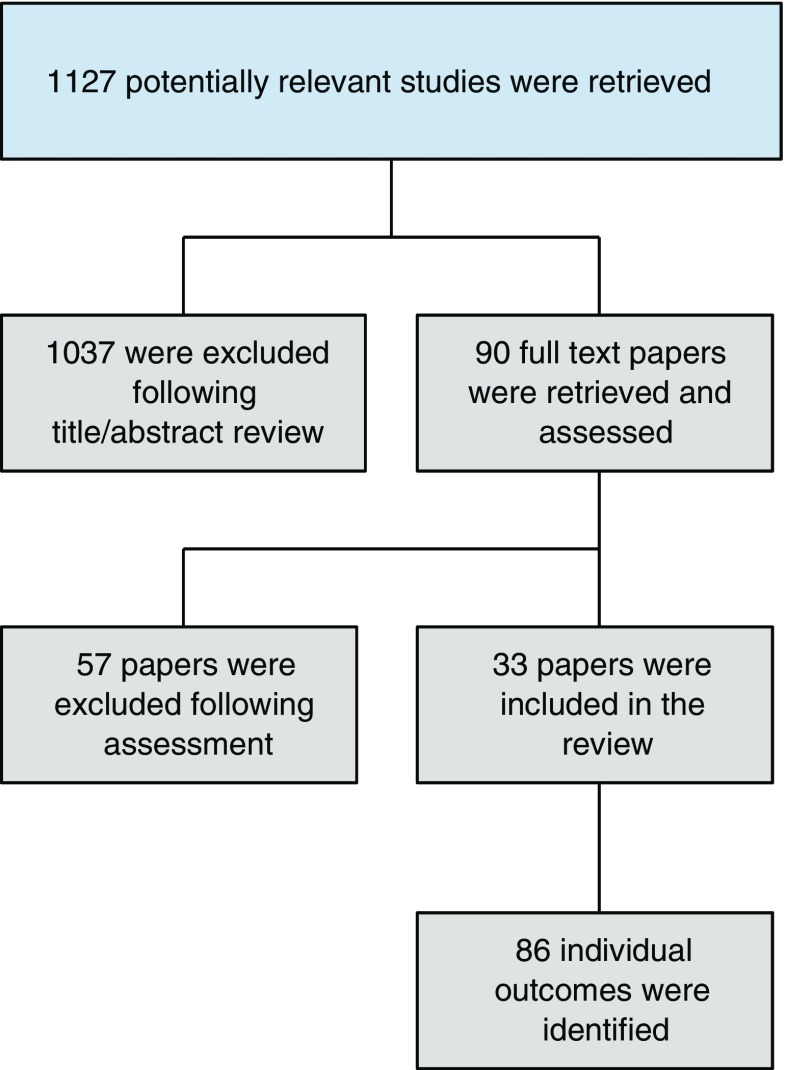
Selection of studies for systematic review

A total of 1127 titles and abstracts were identified. Following review of the title and/or abstracts, 90 full text papers were retrieved and assessed for eligibility. A further 57 papers were excluded following full text assessment, leaving 33 papers in the review (Fig. 1) [3–5, 13–42]. Following data extraction, 86 individual outcomes were identified. These were grouped according to the following domains: measures of pregnancy preparation (*n* = 38), neonatal outcomes (*n* = 32) and maternal outcomes (*n* = 16).

### Delphi study

The 86 outcomes extracted were presented to the participants grouped by domain. There were 151 respondents to the round 1 instrument (74.2% female) with representation from 24 countries and five continents. A total of 72.2% respondents were from Ireland and the UK. Stakeholders were grouped into three categories. Category 1 consisted of adult endocrinologists (*n* = 43, 28.5%), diabetes nurse specialists (*n* = 8, 5.3%) and dietitians (*n* = 2, 1.3%). Category 2 consisted of midwives (*n* = 17, 11.3%) and obstetricians (*n* = 23, 15.2%). Category 3 consisted of women with diabetes (*n* = 20, 13.2%), policy makers (*n* = 1, 0.7%), researchers in the area of diabetes (*n* = 14, 9.3%), advocates on behalf of those with diabetes (*n* = 2, 1.3%) and others (*n* = 21, 13.9%). Those that selected ‘other’ were from a variety of healthcare backgrounds and included GPs, anaesthesiologists and neonatologists. ESM Table 1 outlines the median score for each outcome based on the response to the round 1 instrument. An additional 27 outcomes were suggested by round 1 respondents and included in round 2.

Round 2 participants were asked to rate 113 outcomes as follows: measures of pregnancy preparation (*n* = 51), neonatal outcomes (*n* = 39) and maternal outcomes (*n* = 23). The round 2 instrument was completed by 120 people who had completed round 1 (78.4%). ESM Table 1 outlines the median score for each outcome based on the responses to the round 2 instrument. All outcomes had a median score of ≥4 and, therefore, 113 outcomes were carried forward to round 3.

The round 3 instrument was completed by 101 participants who had completed round 2 (84.2%) and the median score for each outcome is outlined in ESM Table 1. ESM Table 2 outlines the percentage of round 3 participants scoring each outcome as 1–3, 4–6 and 7–9 on the nine point scale. A total of 84 (74.3%) outcomes were classified as ‘consensus in’ and 29 (25.7%) were classified as ‘no consensus’.

### Consensus meeting

The consensus meeting involved 14 stakeholders, a chairperson and two administrators. The stakeholders included two women with type 1 diabetes, five adult endocrinologists, one diabetes nurse specialist, two midwives (of whom, one has type 1 diabetes), two obstetricians and two researchers in the area of diabetes and pregnancy. ESM Table 3 outlines the percentage of participants voting ‘yes’ for each outcome in rounds 1 and 2. Based on the views of the group, several outcomes were rephrased and/or combined. These are described in the ESM.

Voting took place for each outcome in the modified list of outcomes (*n* = 108). Following round 1 voting, 20 outcomes were considered for inclusion in the COS. Following further discussion and round 2 voting, 17 outcomes were selected and agreed on for inclusion in the final COS as presented in Table 1.

**Table 1:** Final COS to be included in all studies of prepregnancy care for women with pregestational diabetes (*n* = 17)

Domain	Outcome
Measures of pregnancy preparation (*n* = 9)	Healthcare professional review prior to conception
	Smoking status at first antenatal visit
	Use of folic acid preconception
	Thyroid function at first antenatal visit
	Use of potentially teratogenic medications at conception
	Gestational age at first antenatal visit
	BMI at first antenatal visit
	BP at first antenatal visit
	First trimester HbA_1c_
Neonatal outcomes (*n* = 6)	Perinatal mortality
	Miscarriage
	Congenital malformation
	Preterm birth
	Large for gestational age
	Small for gestational age
Maternal outcomes (*n* = 2)	Gestational weight gain
	Severe maternal hypoglycaemia in first trimester

## Discussion

In this study, 17 core outcomes were identified and agreed on for inclusion in a COS essential for studies evaluating prepregnancy care for women with pregestational diabetes. These outcomes were grouped into three domains that include measures of pregnancy preparation, neonatal outcomes and maternal outcomes. It is advocated that all trials and other non-randomised studies and audit in this area use this COS with the aim of improving transparency and the ability to compare and combine future studies with greater ease.

The rationale for the development of such a COS is convincing. A recent survey of 788 Cochrane reviews found that 37% of prespecified outcomes were not actually reported [43]. A 2012 systematic review and meta-analysis of prepregnancy care for women with pregestational diabetes noted a wide variety in outcomes reported in the included studies [44], a finding that significantly limits interpretation of the results. Additionally, considering a recent Cochrane review on preconception care for diabetic women advising the need for further high-quality studies in this area [45], it is important that there is guidance on selecting appropriate outcomes for evaluation. This study fills an important gap in the literature, as there is currently no COS for prepregnancy care for women with diabetes.

This study has several strengths. Robust consensus methodology and guidance from the COMET initiative were used to develop the COS [11]. A detailed study protocol was published [6] and the COS-STAR statement was used to ensure clarity and a high standard of reporting [9]. The Delphi process aims to elicit and condense the opinions of many towards consensus. It facilitates a large and international participation, and it allows each participant to have an equal voice in rating and suggesting additional outcomes for consideration. There is a lack of consensus on optimal consensus meeting size and representativeness is assessed on the qualities of the expert panel rather than the numbers [46, 47]. As outlined in our protocol, we included three service users along with a diverse group of experienced healthcare professionals and researchers representing a broad range of viewpoints [6]. The opinion of the participants was that the consensus meeting was collaborative and inclusive. Prior to altering any outcomes from the Delphi study, significant discussion took place with opinions invited from all participants. The downloadable electronic application used for anonymous voting prevented individuals feeling pressurised to vote in a certain way.

A limitation of the systematic review is the inclusion of studies published in the English language only, which may have introduced a selection bias. Following the systematic review, the determination of three domains introduced subjectivity in terms of outcome categorisation. Several independent reviews were taken to reduce this. The study protocol stated that within each domain outcomes would be listed alphabetically [6]. The intent was to avoid weighting of outcomes caused by the order in which they were displayed. In the actual study, related outcomes were presented alongside each other. The SAG felt that this was more appropriate and would encourage participants to consider overlap between outcomes within domains during the scoring process. In relation to the online survey, the authors acknowledge the risk of nonresponse bias. Due to our sampling approach, we do not have an appreciation for the numbers of potential participants who declined to respond; however, approximately one-third of round 1 participants did not continue to complete round 3. In addition, while participants came from a variety of backgrounds and countries of residence, the majority were European and developing countries were not represented. The potential effect of this is not easily measurable but it may limit the generalisability of the study to less affluent areas of the world.

The final number of outcomes included in the COS may be considered relatively large; however, this is related to the nature of prepregnancy care which has potential effects before, during and after pregnancy for two individuals, both mother and child. The authors wish to highlight that in the setting of future randomised controlled trials in this area, we would not expect an inappropriately large number of primary outcomes to be selected, but rather ensure these outcomes are collected and reported during the study. Another potential criticism of this study is that it does not provide outcome definitions. It must be specified that the purpose of the COS is to define ‘what is to be collected’ and not ‘how it is to be collected’. In the field of diabetes and pregnancy, there exists a previously published repository of definitions that may be referenced as required [48]. Finally, it must be acknowledged that many outcomes were carried forward and selected out in the final, consensus meeting phase of the study. During this phase, delegates were chosen to ensure representation from all stakeholder groups and close attention was paid to outcome scores from round 3 of the Delphi prior to excluding any outcome from the COS.

In conclusion, comparisons between studies evaluating prepregnancy care for women with pregestational diabetes are difficult due to inconsistencies in the approach to collecting and reporting data. This is the first study to define a COS in this area. Its goal is that use of this COS will facilitate international collaboration and allow accurate contrasting and combining of findings. This will make it easier to assess the effect of prepregnancy care, accurately inform policy developers and improve evidence-based practice for women with diabetes.

## Electronic supplementary material

ESM (PDF 480 kb)

## Abbreviations

COMET: Core Outcome Measures in Effectiveness Trials Initiative
COS: Core Outcome Set
COS-STAR: Core Outcome Set – STAndards for Reporting
SAG: Study Advisory Group
