# Supplementary material for: A core outcome set for studies evaluating the effectiveness of prepregnancy care for women with pregestational diabetes
Source: Diabetologia. 2017 Apr 13;60(7):1190–6. doi: 10.1007/s00125-017-4277-4 (PMC5487596; doi:10.1007/s00125-017-4277-4)
Supplement: Supplementary file 1 — (PDF 480 kb) [file 125_2017_4277_MOESM1_ESM.pdf]

Electronic Supplementary Material (ESM)

ESM Results

Consensus Meeting

Based on the views of the group, a number of outcomes were rephrased and/or combined. These are as follows: ‘physician review prior to conception’ was rephrased to ‘healthcare professional review prior to conception’; ‘maternal weight at initial antenatal visit’ was combined with ‘body mass index at 1st antenatal visit’; ‘maternal weight at birth of baby’ was combined with ‘gestational weight gain’; ‘systolic blood pressure at 1st antenatal visit’ and ‘diastolic blood pressure at initial antenatal visit’ were combined to form the new outcome of ‘blood pressure at 1st antenatal visit’; ‘HbA_{1c} when first attending prepregnancy care’ and ‘HbA1c at referral to prepregnancy care’ and ‘preconception HbA_{1c}’ were combined to form ‘HbA_{1c} at 1st attendance at prepregancy care’.

ESM Tables

ESM Table 1: Outcomes included in Delphi study and median score for each outcome

	Round 1 (151 respondents) Median Scores	Round 2 (120 respondents) Median Scores	Round 3 (101 Respondents) Median Scores
Measures of Pregnancy Preparation			
Patient attendance at prepregnancy care	9	9	9
Physician review prior to conception	9	9	8
Discontinuation of contraception for purposes of pregnancy	8	8	9
Self-reported planned pregnancy at initial antenatal visit	8	8	7
Attendance rate at prepregnancy care appointments [a]	-	8	8
Physician assessment of level of pregnancy preparation.[a]	-	8	7
Asssessment of maternal health-related quality of life[a]	-	8	7
Duration of prepregnancy care [a]	-	7	7
Patient satisfaction with prepregnancy care [a]	-	8	7
Smoking status during pregnancy	9	9	9
Smoking status at 1st antenatal visit	9	9	9
Use of folic acid preconception	9	9	9
Use of folic acid at 1st antenatal visit	9	9	9
Thyroid function at 1st antenatal visit [a]	-	8	8
Rubella status at 1st antenatal visit [a]	-	9	9
Iron Status at 1st antenatal visit [a]	-	7	7
Vitamin B12 level at 1st antenatal visit [a]	-	7	7
Vitamin D level at initial antenatal visit	7	7	7
Use of potentially teratogenic meds at conception	9	9	9
Alcohol intake during pregnancy	9	9	9
Gestational age at 1st antenatal visit	9	9	9
Maternal weight at initial antenatal visit	9	9	9
Maternal weight at birth of baby	7	8	8
Body mass index at initial antenatal visit	9	9	9
Systolic blood pressure at initial antenatal visit	9	9	9
Diastolic blood pressure at initial antenatal visit	9	9	9
HbA_{1c} when first attending prepregnancy care	9	9	9
HbA_{1c} at referral to prepregnancy care	8	9	8
Preconception HbA_{1c}	9	9	9
HbA1c during pregnancy	9	9	9
1st trimester HbA_{1c}	9	9	9
2nd trimester HbA_{1c}	9	9	9
3rd trimester HbA_{1c}	8	9	9
HbA_{1c} at birth of baby	8	8	8
HbA_{1c} postpartum	7	7	7
HbA_{1c} at 9 weeks	8	8	7
HbA_{1c} at 12 weeks	8	8	8
HbA_{1c} at 14 weeks	7	8	7
HbA_{1c} at 16 weeks	7	7.5	7
HbA_{1c} at 20 weeks	8	8	7
HbA_{1c} at 24 weeks	8	8	7
HbA_{1c} at 26 weeks	7	8	7
HbA_{1c} at 28 weeks	7	8	7
HbA_{1c} at 32 weeks	7	8	7
HbA_{1c} at 36 weeks	8	8	7
Per-trimester fasting and pre-prandial glucose	9	9	9
Per-trimester 90 minute post-prandial glucose	9	8	7
Per-trimester 60 minute post-prandial glucose [a]	-	8	8
Fructosamine level during pregnancy [a]	-	7	6
Patient compliance with glucose monitoring [a]	-	9	9
Patient compliance with medication/insulin regimen [a]	-	9	9
Neonatal Outcomes			

Shoulder dystocia	9	9	9
Clavicular fracture	8.5	9	9
Erb's palsy	9	9	9
Infant respiratory distress syndrome	9	9	9
Need for mechanical ventilation [a]	-	9	9
Neonatal hyperbilirubinemia	8	8	8
Neonatal hyperbilirubinemia requiring treatment [a]	-	8.5	8
Neonatal hypocalcaemia	8	8	8
Apgar score at 1 min	8	9	9
Apgar score at 5 min	9	9	9
Cord Ph at birth [a]	-	8	8
Birth Asphyxia [a]	-	9	9
Neonatal encephalopathy [a]	-	9	9
Method of feeding infant [a]	-	9	9
Livebirth	9	9	9
Stillbirth	9	9	9
Neonatal death	9	9	9
Perinatal mortality	9	9	9
Miscarriage	9	9	9
Termination of pregnancy	9	9	9
Termination for fetal malformation	9	9	9
Termination for non-diabetes associated issue	8	8	7
Congenital malformation	9	9	9
Major congenital malformation	9	9	9
Weeks of gestation at delivery	9	9	9
Preterm birth	9	9	9
Extremely preterm birth	9	9	9
Infant birthweight at delivery	9	9	9
Large for gestational age	9	9	9
Macrosomia	9	9	9
Small for gestational age	9	9	9
Low birthweight	9	9	9
Composite adverse outcome	8	8	9
Admission to neonatal intensive care unit	9	9	9
Admission to routine postnatal ward	8	7	7
Length of stay in neonatal intensive care unit	9	9	9
Neonatal hypoglycaemia	9	9	9
Severe neonatal hypoglycaemia	9	9	9
Neonatal hypoglycaemia requiring intravenous treatment [a]	-	9	9
Maternal Outcomes			
Gestational hypertension	9	9	9
Pre-eclampsia	9	9	9
Mode of birth	9	9	9
Normal vaginal birth	9	9	9
Instrumental birth	8	9	8
Caesarean birth	9	9	9
Planned / elective lower segment caesarean section	8	9	8
Emergency lower segment caesarean section	9	9	9
Trial of labour	8	8	8
Gestational weight gain	9	9	9
Presence of nephropathy	9	9	9
Nephropathy progression	9	9	9
Presence of retinopathy	9	9	9
Retinopathy Progression	9	9	9
Presence of peripheral neuropathy [a]	-	9	9
Peripheral neuropathy progression [a]	-	9	9
Presence of autonomic neuropathy [a]	-	9	9
Severe maternal hypoglycaemia	9	9	9
Maternal mortality	9	9	9
Symphysis pubis dysfunction [a]	-	7	7

Maternal post partum infection [a]	-	8	8
Maternal inpatient admission during pregnancy [a]	-	8	8
Maternal length of stay in hospital during pregnancy and delivery[a]	-	8	8

[a] outcomes added to round 2 and 3 instruments.

ESM Table 2: Percentage of round 3 participants (n=101) scoring each outcome as 1-3, 4-6 or 7-9 on the 9 point scale. [a]

	Score 1-3	Score 4-6	Score 7-9
Measures of Pregnancy Preparation			
Patient attendance at prepregnancy care	0 %	3 %	97.0%
Physician review prior to conception	11.9%	6.9%	77.2%
Discontinuation of contraception for purposes of pregnancy[b]	11.8%	16.8%	69.3%
Self-reported planned pregnancy at initial antenatal visit[b]	12.9%	15.8%	68.3%
Attendance rate at prepregnancy care appointments	4.9%	11.8%	81.1%
Physician assessment of level of pregnancy preparation[b]	10.9%	19.8%	66.3%
Asssessment of maternal health-related quality of life[b]	10.9%	27.7%	59.4%
Duration of prepregnancy care[b]	7.9%	26.7%	63.4%
Patient satisfaction with prepregnancy care[b]	7.9%	21.7%	66.3%
Smoking status during pregnancy	8.9%	0.9%	89.1%
Smoking status at 1st antenatal visit	3%	13.9%	91.1%
Use of folic acid preconception	0%	1%	99.0%
Use of folic acid at 1st antenatal visit	8.9%	3%	87.1%
Thyroid function at 1st antenatal visit[b]	8.9%	14.9%	66.3%
Rubella status at 1st antenatal visit	10.9%	14.9%	73.3%
Iron Status at 1st antenatal visit[b]	110.9%	27.7%	59.4%
Vitamin B12 level at 1st antenatal visit[b]	12.9%	25.7%	59.4%
Vitamin D level at initial antenatal visit[b]	12.9%	31.7%	55.4%
Use of potentially teratogenic meds at conception	0.9%	0%	98.0%
Alcohol intake during pregnancy	1.9%	4%	92.1%
Gestational age at 1st antenatal visit	0.9%	0.9%	98.0%
Maternal weight at initial antenatal visit	8.9%	5.9%	84.9%
Maternal weight at birth of baby	7.9%	12.9%	78.2%
Body mass index at initial antenatal visit	0%	6.9%	91.1%
Systolic blood pressure at initial antenatal visit	0.9%	5.9%	91.1%
Diastolic blood pressure at initial antenatal visit	0.9%	6.9%	90.1%
HbA_{1c} when first attending prepregnancy care	6.9%	3%	89.1%
HbA_{1c} at referral to prepregnancy care	10.9%	12.9%	72.3%
Preconception HbA_{1c}	4%	4%	90.1%
HbA1c during pregnancy	4%	4%	90.1%
1st trimester HbA_{1c}	7.9%	2%	87.1%
2nd trimester HbA_{1c}	8.9%	8.9%	80.1%
3rd trimester HbA_{1c}	8.9%	6.9%	81.2%
HbA_{1c} at birth of baby[b]	12.9%	20.8%	65.3%
HbA_{1c} postpartum[b]	14.9%	21.8%	60.4%
HbA_{1c} at 9 weeks[b]	15.8%	22.8%	61.4%
HbA_{1c} at 12 weeks[b]	13.9%	19.8%	63.4%
HbA_{1c} at 14 weeks[b]	16.8%	2%	81.2%
HbA_{1c} at 16 weeks[b]	16.8%	27.7%	55.5%
HbA_{1c} at 20 weeks[b]	15.8%	26.7%	57.5%
HbA_{1c} at 24 weeks[b]	14.9%	24.9%	60.2%
HbA_{1c} at 26 weeks[b]	16.8%	28.7%	54.5%
HbA_{1c} at 28 weeks[b]	15.8%	25.7%	58.5%
HbA_{1c} at 32 weeks[b]	16.8%	24.9%	58.3%
HbA_{1c} at 36 weeks[b]	15.8%	21.8%	62.4%
Per-trimester fasting and pre-prandial glucose	10.9%	4.9%	82.2%
Per-trimester 90 minute post-prandial glucose[b]	13.9%	16.8%	69.3%
Per-trimester 60 minute post-prandial glucose	10.9%	10.9%	78.2%
Fructosamine level during pregnancy[b]	17.8%	34.7%	47.5%
Patient compliance with glucose monitoring	10.9%	6.9%	79.2%
Patient compliance with medication/insulin regimen	10.9%	4.9%	82.2%
Neonatal Outcomes			
Shoulder dystocia	0%	4%	94.9%
Clavicular fracture	4.9%	9.9%	81.2%

Erb's palsy	4%	5.9%	87.1%
Infant respiratory distress syndrome	3%	3%	91.1%
Need for mechanical ventilation	4.9%	6.9%	85.1%
Neonatal hyperbilirubinemia	3%	15.8%	77.2%
Neonatal hyperbilirubinemia requiring treatment	5.9%	10.9%	81.2%
Neonatal hypocalcaemia	4.9%	15.8%	76.2%
Apgar score at 1 min	4%	8.9%	84.9%
Apgar score at 5 min	1%	3%	94.9%
Cord Ph at birth	6.9%	15.8%	73.3%
Birth Asphyxia	8.9%	4%	83.2%
Neonatal encephalopathy	8.9%	8.9%	79.2%
Method of feeding infant	2%	12.9%	81.2%
Livebirth	2%	0%	94.1%
Stillbirth	2%	1%	95%
Neonatal death	2%	2%	94.9%
Perinatal mortality	6.9%	2%	89.1%
Miscarriage	2%	5.9%	89.1%
Termination of pregnancy	6.9%	15.8%	75.2%
Termination for fetal malformation	8.9%	3%	86.1%
Termination for non-diabetes associated issue	13.9%	15.8%	67.3%
Congenital malformation	1%	2%	93.1%
Major congenital malformation	6.9%	2%	85.1%
Weeks of gestation at delivery	1%	0%	96%
Preterm birth	6.9%	4.9%	86.1%
Extremely preterm birth	8.9%	6.9%	80.2%
Infant birthweight at delivery	0%	3%	94.9%
Large for gestational age	3%	5.9%	88.1%
Macrosomia	7.9%	9.9%	79.2%
Small for gestational age	4%	3%	87.1%
Low birthweight	9.9%	8.9%	77.2%
Composite adverse outcome	7.9%	9.9%	75.2%
Admission to neonatal intensive care unit	0%	2%	96%
Admission to routine postnatal ward	9.9%	15.8%	46.5%
Length of stay in neonatal intensive care unit[b]	4.9%	10.9%	80.1%
Neonatal hypoglycaemia	0.9%	2%	94.9%
Severe neonatal hypoglycaemia	9.9%	7.9%	82.2%
Neonatal hypoglycaemia requiring intravenous treatment	7.9%	12.9%	85.1%
Maternal Outcomes			
Gestational hypertension	2%	2%	95%
Pre-eclampsia	2%	2%	95%
Mode of birth	2%	7.9%	87.1%
Normal vaginal birth	8.9%	12.9%	74.9%
Instrumental birth	8.9%	10.9%	77.2%
Caesarean birth	8.9%	9.9%	78.2%
Planned / elective lower segment caesarean section	9.9%	10.9%	75.2%
Emergency lower segment caesarean section	9.9%	5.9%	80.2%
Trial of labour[b]	9.9%	15.8%	69.3%
Gestational weight gain	0.9%	6.9%	86.1%
Presence of nephropathy	8.9%	4.9%	84.9%
Nephropathy progression	0.9%	2%	94.9%
Presence of retinopathy	8.9%	4%	83.2%
Retinopathy Progression	0.9%	3%	94.9%
Presence of peripheral neuropathy	9.9%	7.9%	78.2%
Peripheral neuropathy progression	4%	6.9%	85.1%
Presence of autonomic neuropathy	9.9%	6.9%	79.2%
Severe maternal hypoglycaemia	0%	2%	96%
Maternal mortality	0%	0.9%	96%
Symphysis pubis dysfunction[b]	9.9%	29.7%	60.4%
Maternal post partum infection[b]	10.9%	18.8%	67.3%
Maternal inpatient admission during pregnancy	6.9%	15.8%	74.9%

Maternal length of stay in hospital during pregnancy and delivery[b]	9.9%	17.8%	69.3%

[a] Instances of cumulative scores not reaching 100% result from participants leaving an outcome undefined.
[b] Indicates that the outcome was classified as “no consensus”. Remaining outcomes were classified as “consensus in”.

ESM Table 3: % consensus meeting participants voting "yes" to include each outcome in the final core outcome set

	Round 1 Voting (% voting yes)	Round 2 Voting (% voting yes)
Measures of Pregnancy Preparation		
Patient attendance at prepregnancy care	78.6%	0%
Healthcare professional review prior to conception[a]	71.4%	100%
Discontinuation of contraception for purposes of pregnancy	7.1%	-
Self-reported planned pregnancy at initial antenatal visit	14.3%	-
Attendance rate at prepregnancy care appointments	7.1%	-
Physician assessment of level of pregnancy preparation	0%	-
Asssessment of maternal health-related quality of life	14.3%	-
Duration of prepregnancy care	7.1%	-
Patient satisfaction with prepregnancy care	14.3%	-
Smoking status during pregnancy	42.9%	-
Smoking status at 1st antenatal visit[a]	85.7%	100%
Use of folic acid preconception[a]	100%	100%
Use of folic acid at 1st antenatal visit	35.7%	-
Thyroid function at 1st antenatal visit[a]	71.4%	70%
Rubella status at 1st antenatal visit	57.1%	-
Iron Status at 1st antenatal visit	14.3%	-
Vitamin B12 level at 1st antenatal visit	7.1%	-
Vitamin D level at initial antenatal visit	28.6%	-
Use of potentially teratogenic medications at conception[a]	92.9%	100%
Alcohol intake during pregnancy	35.7%	-
Gestational age at 1st antenatal visit[a]	85.7%	100%
Body mass index at 1st antenatal visit[a]	100%	100%
Blood pressure at 1st antenatal visit[a]	92.9%	100%
HbA_{1c} at first attendance at prepregnancy care	92.9%	60%
HbA_{1c} during pregnancy	7.1%	-
1st trimester HbA_{1c}[a]	100%	100%
2nd trimester HbA_{1c}	21.4%	-
3rd trimester HbA_{1c}	14.3%	-
HbA_{1c} at birth of baby	14.3%	-
HbA_{1c} postpartum	14.3%	-
HbA_{1c} at 9 weeks	0%	-
HbA_{1c} at 12 weeks	0%	-
HbA_{1c} at 14 weeks	0%	-
HbA_{1c} at 16 weeks	0%	-
HbA_{1c} at 20 weeks	0%	-
HbA_{1c} at 24 weeks	0%	-
HbA_{1c} at 26 weeks	0%	-
HbA_{1c} at 28 weeks	0%	-
HbA_{1c} at 32 weeks	0%	-
HbA_{1c} at 36 weeks	0%	-
Per-trimester fasting and pre-prandial glucose	14.30%	-
Per-trimester 90 minute post-prandial glucose	0%	-
Per-trimester 60 minute post-prandial glucose	0%	-
Fructosamine level during pregnancy	0%	-
Patient compliance with glucose monitoring	35.7%	-
Patient compliance with medication/insulin regimen	42.9%	-
Neonatal Outcomes		
Shoulder dystocia	35.7%	-
Clavicular fracture	7.1%	-
Erb's palsy	7.1%	-
Infant respiratory distress syndrome	21.4%	-
Need for mechanical ventilation	7.1%	-
Neonatal hyperbilirubinemia	0%	-
Neonatal hyperbilirubinemia requiring treatment	7.1%	-

Neonatal hypocalcaemia	0%	-
Apgar score at 1 min	14.3%	-
Apgar score at 5 min	28.6%	-
Cord Ph at birth	7.1%	-
Birth Asphyxia	14.3%	-
Neonatal encephalopathy	7.1%	-
Method of feeding infant	42.9%	-
Livebirth	64.3%	-
Stillbirth	50%	-
Neonatal death	42.9%	-
Perinatal mortality[a]	92.9%	100%
Miscarriage[a]	85.7%	100%
Termination of pregnancy	57.1%	-
Termination for fetal malformation	50%	-
Termination for non-diabetes associated issue	7.1%	-
Congenital malformation[a]	85.7%	100%
Major congenital malformation	57.1%	
Weeks of gestation at delivery	41.7%	-
Preterm birth[a]	75%	90%
Extremely preterm birth	75%	0%
Infant birthweight at delivery	41.7%	-
Large for gestational age[a]	75%	90%
Macrosomia	25%	-
Small for gestational age[a]	75%	80%
Low birthweight	0%	-
Composite adverse outcome	8.4%	-
Admission to neonatal intensive care unit	66.7%	-
Admission to routine postnatal ward	8.4%	-
Length of stay in neonatal intensive care unit	58.3%	-
Neonatal hypoglycaemia	41.7%	-
Severe neonatal hypoglycaemia	41.7%	-
Neonatal hypoglycaemia requiring intravenous treatment	25%	-
Maternal Outcomes		
Gestational hypertension	41.7%	-
Pre-eclampsia	50%	-
Mode of birth	8.4%	-
Normal vaginal birth	25%	-
Instrumental birth	0%	-
Caesarean birth	50%	-
Planned / elective lower segment caesarean section	25%	-
Emergency lower segment caesarean section	41.7%	-
Trial of labour	0%	-
Gestational weight gain[a]	83.3%	100%
Presence of nephropathy	8.4%	-
Nephropathy progression	25%	-
Presence of retinopathy	8.4%	-
Retinopathy Progression	25%	-
Presence of peripheral neuropathy	0%	-
Peripheral neuropathy progression	0%	-
Presence of autonomic neuropathy	0%	-
Severe maternal hypoglycaemia in 1st trimester[a]	100%	100%
Maternal mortality	66.7%	-
Symphysis pubis dysfunction	0%	-
Maternal post partum infection	0%	-
Maternal inpatient admission during pregnancy	25%	-
Maternal length of stay in hospital during pregnancy and delivery	8.4%	-

[a]outcome included in final core outcome set.
